# Thinking outside the curve, part I: modeling birthweight distribution

**DOI:** 10.1186/1471-2393-10-37

**Published:** 2010-07-28

**Authors:** Richard Charnigo, Lorie W Chesnut, Tony LoBianco, Russell S Kirby

**Affiliations:** 1 Departments of Statistics and Biostatistics University of Kentucky Lexington, KY 40506-0027, USA; 2 Department of Epidemiology University of Kentucky Lexington, KY 40536-0003, USA; 3 Interdisciplinary Human Development Institute University of Kentucky Lexington, KY 40506-0051, USA; 4 Department of Community and Family Health University of South Florida Tampa, FL 33612, USA

## Abstract

**Background:**

Greater epidemiologic understanding of the relationships among fetal-infant mortality and its prognostic factors, including birthweight, could have vast public health implications. A key step toward that understanding is a realistic and tractable framework for analyzing birthweight distributions and fetal-infant mortality. The present paper is the first of a two-part series that introduces such a framework.

**Methods:**

We propose describing a birthweight distribution via a normal mixture model in which the number of components is determined from the data using a model selection criterion rather than fixed *a priori*.

**Results:**

We address a number of methodological issues, including how the number of components selected depends on the sample size, how the choice of model selection criterion influences the results, and how estimates of mixture model parameters based on multiple samples from the same population can be combined to produce confidence intervals. As an illustration, we find that a 4-component normal mixture model reasonably describes the birthweight distribution for a population of white singleton infants born to heavily smoking mothers. We also compare this 4-component normal mixture model to two competitors from the existing literature: a contaminated normal model and a 2-component normal mixture model. In a second illustration, we discover that a 6-component normal mixture model may be more appropriate than a 4-component normal mixture model for a general population of black singletons.

**Conclusions:**

The framework developed in this paper avoids assuming the existence of an interval of birthweights over which there are no compromised pregnancies and does not constrain birthweights within compromised pregnancies to be normally distributed. Thus, the present framework can reveal heterogeneity in birthweight that is undetectable via a contaminated normal model or a 2-component normal mixture model.

## Background

The impact of birthweight on perinatal mortality and morbidity has been debated for decades [1-11]. Although advances in maternal and perinatal care have reduced overall mortality, infants with very low birthweights (1000-1500 g; VLBW) and extremely low birthweights (<1000 g; ELBW) remain at high risk. These infants require more intensive utilization of health resources, at increased costs relative to normal birthweight (NBW; 2500-4000 g) infants [12-14]. Even infants of moderately low birthweight (1500-2500 g; MLBW) and high birthweight (>4000 g; HBW) have elevated mortality and morbidity [15,16]. Greater epidemiologic understanding of the relationships among fetal-infant mortality and its prognostic factors, including birthweight, could have vast public health implications. A key step toward that understanding is a realistic yet tractable framework for analyzing birthweight distribution and fetal-infant mortality.

Simple bell curves are inadequate characterizations of birthweight distributions [17,11-20]. Wilcox and Russell proposed a contaminated normal model, in which a predominant normal distribution accounts for most birthweights while a contaminating residual distribution yields most VLBW and ELBW cases [21]. The residual distribution does not have a specific structure and, in particular, is not normal. The contaminated normal model was later extended by Umbach and Wilcox to accommodate two residual distributions, one yielding excess births in the left tail and the other in the right tail [22].

Gage and Therriault took a different approach, employing a 2-component normal mixture model [23]. A primary normal distribution accounts for most birthweights, while a secondary normal distribution is linked not only to most VLBW and ELBW cases but also to many HBW cases. The 2-component normal mixture (resp., contaminated normal model) dichotomizes birthweights: those arising from the primary distribution (resp., predominant distribution) are conceptualized as reflecting ordinary fetal development, while the rest are considered to signify compromised fetal development [24]. Gage also formulated a parametric mixtures of logistic regressions (PMLR) technique to evaluate heterogeneity in mortality associated with this dichotomy [24].

While the aforementioned works demonstrate great insight, their statistical models have some limitations. In particular, the number of constituent distributions (predominant, residual, primary, secondary) is fixed *a priori*. If a constituent distribution can signify compromised fetal development [24], perhaps different biological mechanisms for compromised fetal development warrant a model with more than two or three constituent distributions. Likewise, perhaps more than two or three birthweight-specific mortality curves are needed to describe heterogeneity in mortality.

The present paper is the first in a two-part series that introduces a new framework for modeling birthweight distribution and fetal-infant mortality. We propose a normal mixture model for birthweight distribution in which the number of components is not fixed *a priori *but rather determined from the data using the Flexible Information Criterion (FLIC) (Pilla and Charnigo, Consistent estimation and model selection in semiparametric mixtures, submitted) or another model selection technique [25,26]. In the companion paper, we show how to estimate birthweight-specific mortality within each component using a generalization of PMLR [24] and how to compare mortality across components within a single population or across populations within a single component. In both papers, we seek statistical models that provide an empirically reasonable fit to the data. However, the goal is not to find good fitting models for their own sake. Rather, such models may lead to better assessments of mortality.

## Results

### 1. Pragmatics for mixture modeling

#### a. Finite normal mixture models

Many phenomena cannot be accurately described via a normal distribution. When no other commonly used probability distribution seems appropriate, a finite normal mixture model is often reasonable. We now briefly describe the model. Readers interested in theoretical developments may consult references [27-30] and works cited therein.

Let *f*(*x;μ,σ*) denote the probability density for the normal distribution with mean *μ *and standard deviation *σ*. A finite normal mixture model with *k *components has probability density(1)

A common way to interpret Equation (1) is to imagine that the full population consists of *k *subpopulations. The proportion of individuals in the full population belonging to subpopulation *j *is *p_j_*. In subpopulation *j*, measurements are normally distributed with mean *μ_j _*and standard deviation *σ_j_*.

The mixture components may or may not represent subpopulations with obvious biological definitions outside the statistical model. For example, in a 2-component normal mixture describing birthweights for white singletons in the United States, there is not an obvious biological characterization for the two components: we may say that the component with the smaller mean reflects compromised pregnancies, but we cannot immediately attribute the compromised pregnancies to a specific biological mechanism.

Ideally, modeling with finite normal mixtures may lead to discoveries of subpopulations with biological definitions that were not immediately obvious, although the mixture components themselves may still only be approximations to such subpopulations.

#### b. Order selection and the flexible information criterion

Equation (1) may be an imperfect description of real data regardless of *k*, but with *k *sufficiently large the description may be adequate to address a problem of scientific interest. Conversely, if *k *is too large, the model may become unwieldy. Hence, a researcher with real data must confront the problem of "order selection" (i.e., choosing an appropriate number of components).

Let *M *denote the maximum number of components that a researcher is willing to accept. For 1 ≤ *m *≤ *M*, let *L_m _*denote the maximum value of the likelihood attainable by an *m*-component normal mixture. The Akaike Information Criterion (AIC) [25], Bayesian Information Criterion (BIC) [26], and Flexible Information Criterion (FLIC) (Pilla and Charnigo, Consistent estimation and model selection in semiparametric mixtures, submitted) are(2)(3)(4)

Above, (3*m *- 1) is the number of free parameters in an *m*-component normal mixture. Also, *n *denotes the sample size, *δ *the average fraction of within-component variability to total variability over the *M *normal mixtures fitted by maximum likelihood, and *B*(*n,δ*) a bivariate function taking values between 0 and 1 (Pilla and Charnigo, Consistent estimation and model selection in semiparametric mixtures, submitted). The criteria balance fidelity to the observed data against model complexity; models are preferred for which the criteria are smaller. Note that *m *indexes normal mixtures being judged by the criteria, while *k *pertains to a normal mixture that has been adopted for data analysis.

The FLIC is distinguished from the AIC and BIC in that its penalty term  is determined not only by the sample size but also by the configuration of data points: a configuration suggesting greater heterogeneity allows a model with more components to be selected. The penalty term of the FLIC also depends on *M*, so that a researcher must specify *M*. In analyzing birthweight data, we fix *M *= 7 since having too many components would impede inference about mortality risk. The FLIC and AIC perform well for small samples, while the FLIC and BIC are better for large samples, so we prefer to rely on the FLIC (Pilla and Charnigo, Consistent estimation and model selection in semiparametric mixtures, submitted).

#### c. Computational procedures

To employ the FLIC, we must obtain maximum likelihood estimates of the proportions, means, and standard deviations in all finite normal mixture models under consideration. For models with more than one component, numerical optimization procedures must be used. We apply the expectation maximization (EM) algorithm to obtain preliminary estimates [31], followed by the optimization (optim) procedure in version 2.3.1 of R (R Foundation for Statistical Computing, Vienna, Austria, 2006) to acquire final estimates. Our R code is available upon written request to the corresponding author. See Section I of [Additional file 1] for details on using EM and optim, including initial value specification.

### 2. Analyzing birthweight data with the FLIC

#### a. A FLIC-selected model and competitors

To exemplify use of the FLIC, we draw a random sample of size 50,000 from the 202,849 white singletons who were born (or experienced fetal death) from 2000 to 2002 and whose mothers smoked heavily (at least twenty cigarettes per day). Since records with birthweights less than 500 grams or gestational ages less than 22 weeks were not consistently documented [32], we require infants in our sample to have known gestational ages of at least 22 weeks and birthweights between 500 and 5500 grams. The data source is the National Center for Health Statistics (NCHS) Public-Use Perinatal Mortality Data Files.

**Figure 1:**
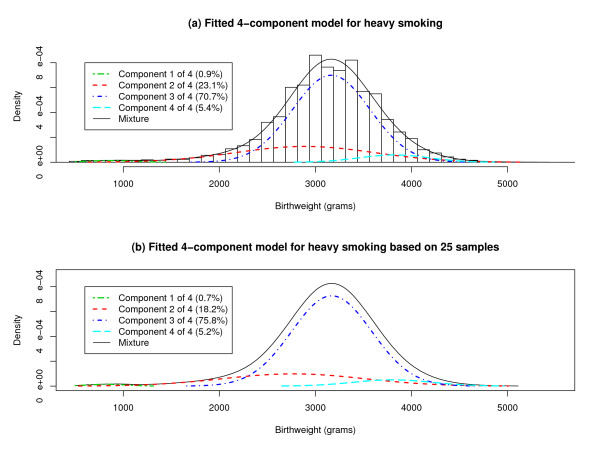
**Four-Component Mixture Models for Birthweight Distribution**. (a) A 4-component normal mixture model for birthweight distribution, with parameters estimated using a single sample of size 50000 from the population of white singletons born to heavily smoking mothers, is shown superimposed against a histogram of the 50000 birthweights. (b) A 4-component normal mixture model, with parameters estimated by combining the results for 25 samples of size 50000, is shown.

The FLIC selects a 4-component model (Figure 1a),(5)

Component 3 is loosely analogous to the predominant distribution in the contaminated normal model [22] and the primary distribution in the 2-component model [23]. Component 1 in the 4-component model includes ELBW and VLBW cases, component 2 contains mostly MLBW and NBW cases but also some VLBW and HBW cases, and component 4 comprises NBW and HBW cases.

Next we fit the contaminated normal and 2-component models to the same data set. For the contaminated normal model, we take the bin width to be 200 grams and use the BIC to select the number of contaminated bins [22]. Approximately 2.5% of cases are assigned to the lower residual distribution (threshold: 1700 grams), 97.5% to the predominant distribution (estimated mean and standard deviation, 3168 and 488 grams), and less than 1 in 8700 to the upper residual distribution (threshold: 5300 grams). Regarding the 2-component model, approximately 88.0% of cases are assigned to the primary distribution (estimated mean and standard deviation, 3186 and 458 grams) and 12.0% to the secondary distribution (estimated mean and standard deviation, 2617 and 951 grams).

**Figure 2:**
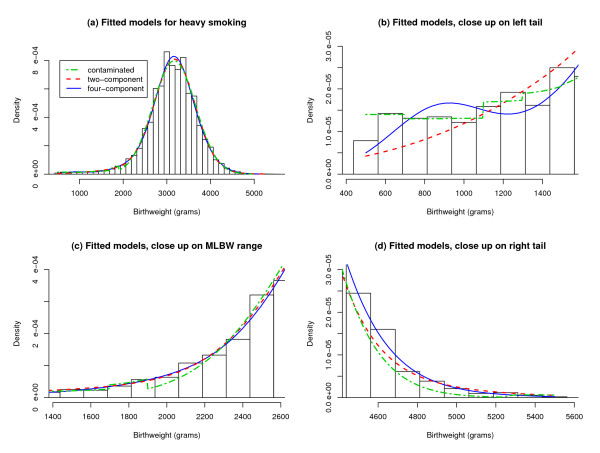
**Competing Models for Birthweight Distribution**. (a) A contaminated normal model, a 2-component normal mixture model, and a 4-component normal mixture model are compared. The results are based on a single sample of size 50000 from the population of white singletons born to heavily smoking mothers. (b) to (d) Close-up views of the competing models are displayed.

The fitted contaminated normal, 2-component, and 4-component models are compared in Figure 2. The contaminated normal model fits the ELBW and VLBW data nicely but exhibits artifacts at the thresholds of 1700 and 5300 grams; the contaminated normal model also understates the HBW data. The 2-component model provides a good fit at most birthweights but severely understates the ELBW data. The 4-component model avoids these weaknesses but has an exaggerated peak near the component 1 mean.

#### b. Reproducibility of order selection

In the preceding example, the selection of a 4-component model was based on a specific sample of 50,000 white singletons whose mothers smoked heavily. If we draw another sample of size 50,000, will the FLIC express the same preference?

We can address this question by drawing *N_rep _*samples of size 50,000 with replacement and applying the FLIC to each sample. Here "with replacement" means that an infant can appear in more than one sample, not that an infant can appear twice in the same sample. The frequency with which the FLIC prefers a 4-component model indicates the reproducibility of order selection.

Table 1 shows the verdicts of the FLIC and other criteria for *N_rep _*= 25 samples of size 50,000. The FLIC prefers a 4-component model for 22 out of 25 samples; for the other three samples, the FLIC narrowly prefers a 6-component model. The verdicts of the BIC match those of the FLIC. The AIC is equivocal between 6-component and 7-component models. Table 1 also identifies the preferences of the FLIC for sample sizes smaller than 50,000. The tendency to favor simpler models at smaller sample sizes can be understood by analogy to a hypothesis test. Imagine testing a null hypothesis that there are two components against an alternative hypothesis that there are more than two components: as the sample size decreases, the power to reject a false null hypothesis also decreases.

**Table 1:** Preferences of Model Selection Criteria on Real Data

Number of Components	FLIC 5000	BIC 5000	AIC 5000	FLIC 10000	BIC 10000	AIC 10000
1	0	0	0	0	0	0

2	20	21	1	8	11	0

3	5	4	4	5	5	0

4	0	0	14	12	9	15

5	0	0	1	0	0	1

6	0	0	4	0	0	8

7	0	0	1	0	0	1

**Number of Components**	**FLIC** **25000**	**BIC** **25000**	**AIC** **25000**	**FLIC** **50000**	**BIC** **50000**	**AIC** **50000**

1	0	0	0	0	0	0

2	0	0	0	0	0	0

3	0	0	0	0	0	0

4	25	25	5	22	22	1

5	0	0	1	0	0	0

6	0	0	10	3	3	6

7	0	0	9	0	0	18

The columns "FLIC 5000", "BIC 5000", and "AIC 5000" contain the preferences of the three model selection criteria for the number of components in a normal mixture model for birthweight distribution, based on 25 samples of size 5000 from the population of white singletons born to heavily smoking mothers. The next nine columns correspond to sample sizes of 10000, 25000, and 50000.

#### c. Uncertainty in parameter estimation

Although we may be comfortable using a 4-component model for the birthweights of white singletons whose mothers smoked heavily, Equation (5) does not convey the uncertainty in the parameter estimates for that model.

To assess uncertainty in parameter estimation, we fit *k*-component models using each of *N_rep _*samples of equal size; in our example, *k *= 4 and there are *N_rep _*= 25 samples of size 50,000. Let *θ *represent a parameter of interest, such as *μ*_3_, and let  represent estimates of *θ *from the *N_rep _*samples. With  denoting the "meta-sample" mean of  and serving as an overall estimate of *θ*, and with  denoting the corresponding standard deviation, we can define a confidence interval via(6)

If  were normally distributed with expected value *θ*, then for 95% confidence we should choose *C *as the upper .025 quantile of the standard normal distribution (or of a T distribution); in the absence of normality, to be conservative we could choose  based on Chebychev's inequality [33]. However, not even *C *= 5.0 yields a coverage probability of 95% (see Section 3b of Results). There are two problems.

First, mixture model parameter estimates may have non-negligible bias; the expected value of  may not be close to *θ*. Second, when each of the *N_rep _*samples constitutes a large fraction of the underlying population,  are not independent due to the large overlaps among the *N_rep _*samples.

The first problem can be addressed by modifying Equation (6) to(7)

where  denotes the estimated absolute value of the bias [34]. Our approach to acquiring  is simulation-based. We simulate a birthweight data set from , where  are the overall estimates of their respective parameters, and then compare  to its own estimate  arising from the simulated data set: the "drift" from  to  should mirror the drift from *θ *to . However, since relying on a single simulated data set seems precarious, we define  as the average value of  over five simulated data sets.

The second problem can be resolved by choosing the value of *C *according to the fraction of the underlying population that each of the *N_rep _*samples constitutes. Let *C*_0 _denote the value of *C *that would be chosen if this fraction were negligibly small, and let *C_φ _*denote the value that would be chosen if this fraction were equal to *φ*, a positive number less than 1. In Section II of [Additional file 1], we show that(8)

Section II of [Additional file 1] also explains why we sample with replacement, why we sample instead of using the full population, and how to compare parameters within and between populations.

Table 2 lists overall estimates and confidence intervals for parameters in a 4-component model for the birthweights of white singletons born to heavily-smoking mothers, using Equations (7) and (8) with the same *N_rep _*= 25 samples of size 50,000 in Table 1, *C*_0 _= 2.5 (see Section 3b of Results), and *φ *= .2465 = 50,000/202,849. Figure 1b displays the mixture model implied by the overall estimates in Table 2. Section III of [Additional file 1] examines how the overall estimates and confidence intervals change when the sample size is less than 50,000.

**Table 2:** Estimating Parameters in a Four-Component Mixture Model

Quantity	p_1_	p_2_	p_3_	p_4_
[average of 25 estimates]	.007	.182	.758	.052

[standard deviation of 25 estimates]	.001	.039	.037	.008

[bias adjustment]	.001	.041	.032	.009

Confidence interval	(.005, .010)	(.092, .272)	(.681, .836)	(.033, .071)

Quantity	μ_1_	μ_2_	μ_3_	μ_4_

[average of 25 estimates]	832	2772	3170	3804

[standard deviation of 25 estimates]	46	103	7	25

[bias adjustment]	34	80	9	38

Confidence interval	(741, 924)	(2565, 2979)	(3152, 3187)	(3735, 3873)

Quantity	σ_1_	σ_2_	σ_3_	σ_4_

[average of 25 estimates]	210	740	417	413

[standard deviation of 25 estimates]	28	23	10	38

[bias adjustment]	30	23	7	46

Confidence interval	(146, 274)	(688, 792)	(398, 436)	(321, 506)

Parameters in a 4-component normal mixture model for birthweight distribution are estimated, based on 25 samples of size 50000 from the population of white singletons born to heavily smoking mothers. Interval estimates are constructed using Equations (7) and (8) with *C*_0 _= 2.5 and *φ *= .2465.

### 3. Further illustrations

#### a. Simulation study on model selection

**Figure 3:**
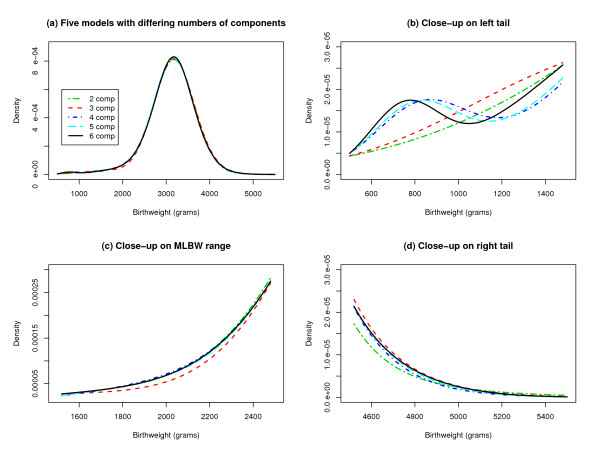
**Mixture Models in Simulation Studies**. (a) Probability densities for the normal mixture models used in our simulation studies are compared. (b) to (d) Close-up views of the probability densities are displayed.

For our first simulation study we generated 25 nonoverlapping data sets of size 5000 from designs A through E in Table 3; see also Figure 3. Designs A through E represent the fitted 2- through 6-component models derived from the 25 samples of size 50,000 in Table 1. Values in the data sets less than 500 or greater than 5500 were discarded since the 2- through 6-component models were meant to mimic a birthweight distribution; new values were drawn as needed to complete the data sets. We assessed how often the FLIC, BIC, and AIC recovered the correct number of components. This was repeated for data sets of different sizes up to 100,000.

**Table 3:** Mixture Models in Simulation Studies

Design	Description	Mixture Density
A	2 components	.120 *f(x;*2601,947) + .880 *f(x;*3186,457)

B	3 components	.041 *f(x;*1673,617) + .871 *f(x;*3162,455) + .088 *f(x;*3537,575)

C	4 components	.007 *f(x;*832,210) + .182 *f(x;*2772,740) + .758 *f(x;*3170,417) + .052 *f(x;*3804,413)

D	5 components	.007 *f(x;*803,193) + .086 *f(x;*2323,631) + .678 *f(x;*3114,419) + .214 *f(x;*3441,441) + .014 *f(x;*4142,428)

E	6 components	.006 *f(x;*752,160) + .032 *f(x;*1737,471) + .268 *f(x;*2829,442) + .586 *f(x;*3215,373) + .099 *f(x;*3762,353) + .010 *f(x;*4337,387)

Probability densities for normal mixture models used in our simulation studies are specified.

As shown in Table 4, the FLIC and BIC consistently returned the correct answer with the 2-component model at a sample size of 5000, the 3-component model at a sample size of 10,000, and the 4-component model at a sample size of 25,000. The FLIC and BIC did not consistently return the correct answer for the 5-component or 6-component model at any sample size, although they occasionally detected components 5 and 6 at a sample size of 100,000. The AIC was erratic.

**Table 4:** Preferences of Model Selection Criteria in Simulation Studies

True Model	Sample size	FLIC preferences						BIC preferences						AIC preferences					
		
		2	3	4	5	6	7	2	3	4	5	6	7	2	3	4	5	6	7
2	5000	25	0	0	0	0	0	25	0	0	0	0	0	7	9	1	3	4	1
	
	10000	25	0	0	0	0	0	25	0	0	0	0	0	2	12	7	2	2	0
	
	25000	20	5	0	0	0	0	21	4	0	0	0	0	0	5	4	8	4	4
	
	50000	5	19	1	0	0	0	5	19	1	0	0	0	0	0	2	6	10	7
	
	100000	0	20	3	1	1	0	0	20	3	1	1	0	0	0	0	0	6	19

3	5000	19	6	0	0	0	0	19	6	0	0	0	0	1	18	5	1	0	0
	
	10000	1	24	0	0	0	0	1	24	0	0	0	0	0	16	6	1	2	0
	
	25000	0	25	0	0	0	0	0	25	0	0	0	0	0	9	4	3	6	3
	
	50000	0	24	1	0	0	0	0	24	1	0	0	0	0	0	2	1	15	7
	
	100000	0	17	3	4	1	0	0	17	3	4	1	0	0	0	0	3	7	15

4	5000	18	2	5	0	0	0	20	1	4	0	0	0	0	1	20	1	2	1
	
	10000	10	1	14	0	0	0	10	1	14	0	0	0	0	1	24	0	0	0
	
	25000	0	0	25	0	0	0	0	0	25	0	0	0	0	0	24	0	1	0
	
	50000	0	0	25	0	0	0	0	0	25	0	0	0	0	0	6	14	3	2
	
	100000	0	0	25	0	0	0	0	0	25	0	0	0	0	0	0	8	10	7

5	5000	21	2	2	0	0	0	22	1	2	0	0	0	0	2	12	9	2	0
	
	10000	9	3	12	1	0	0	9	3	12	1	0	0	0	0	15	9	1	0
	
	25000	0	0	25	0	0	0	0	0	25	0	0	0	0	0	8	10	6	1
	
	50000	0	0	25	0	0	0	0	0	25	0	0	0	0	0	0	10	13	2
	
	100000	0	0	15	10	0	0	0	0	15	10	0	0	0	0	0	6	10	9

6	5000	24	1	0	0	0	0	24	1	0	0	0	0	0	3	7	10	2	3
	
	10000	9	2	13	1	0	0	10	2	13	0	0	0	0	0	12	4	8	1
	
	25000	0	0	23	2	0	0	0	0	23	2	0	0	0	0	0	2	19	4
	
	50000	0	0	24	0	1	0	0	0	25	0	0	0	0	0	0	0	15	10
	
	100000	0	0	10	7	8	0	0	0	11	6	8	0	0	0	0	0	10	15

The row with "True Model" = 2 and "Sample size" = 5000 contains the preferences of the three model selection criteria for the number of components, based on 25 samples of size 5000 simulated from a 2-component normal mixture model. Other rows correspond to a different sample size and/or underlying number of components.

At larger sample sizes, the FLIC and BIC routinely claimed a third (non-existent) component for the 2-component model. We attribute this to the removal of values less than 500 or greater than 5500, after which the 2-component model was, strictly speaking, no longer a normal mixture but rather a truncated normal mixture.

#### b. Simulation study on calibrating confidence intervals

For our second simulation study we generated 25 overlapping data sets of size 50,000 from design C in Table 3, the degree of overlap consistent with a population of 200,000. For each of various *C *between 2.0 and 5.0, we used Equation (7) to form confidence intervals for the mixture parameters *p*_1_, *p*_2_, *p*_3_, *p*_4_, *μ*_1_, *μ*_2_, *μ*_3_, *μ*_4_, *σ*_1_, *σ*_2_, *σ*_3_, *σ*_4_. We recorded how many of the mixture parameters were contained in their respective confidence intervals. This was repeated nine more times, and we tabulated how many of the 120 = 12 × 10 confidence intervals contained their targets. Confidence intervals were also formed using Equation (6) for comparative purposes. The above steps were repeated with overlapping data sets consistent with a population of 1,000,000 and with nonoverlapping data sets consistent with an effectively infinite population.

The results are summarized in Table 5. With an effectively infinite population, only 81.7% of the confidence intervals formed using Equation (6) contained their targets at *C *= 5.0. The confidence intervals formed using Equation (7) contained their targets 95.0% of the time at *C *= 2.5. The adjustment suggested by Equation (8) appears reasonable: *φ *= .05 = 50,000/1,000,000 and *N_rep _*= 25 yield *C_φ _*= 1.315 *C*_0_, which accords with the 95.8% capture of mixture parameters at *C *= 3.5 ≈ 1.315 × 2.5 with a population of 1,000,000.

**Table 5:** Confidence Interval Coverage Probabilities in Simulation Studies

*C*	Population Size	Bias adjustment included	Bias adjustment omitted
		
		Number & Percentage of Intervals Containing Targets (Mixture parameters)	Number & Percentage of Intervals Containing Targets (Mixture parameters)
2.0	200,000	106 (88.3)	50 (41.7)
	
	1,000,000	110 (91.7)	66 (55.0)
	
	Infinite	105 (87.5)	64 (53.3)

2.5	200,000	110 (91.7)	61 (50.8)
	
	1,000,000	112 (93.3)	75 (62.5)
	
	Infinite	114 (95.0)	74 (61.7)

3.0	200,000	110 (91.7)	67 (55.8)
	
	1,000,000	112 (93.3)	80 (66.7)
	
	Infinite	115 (95.8)	83 (69.2)

3.5	200,000	111 (92.5)	68 (56.7)
	
	1,000,000	115 (95.8)	83 (69.2)
	
	Infinite	118 (98.3)	88 (73.3)

4.0	200,000	112 (93.3)	69 (57.5)
	
	1,000,000	118 (98.3)	85 (70.8)
	
	Infinite	118 (98.3)	91 (75.8)

4.5	200,000	113 (94.2)	74 (61.7)
	
	1,000,000	118 (98.3)	87 (72.5)
	
	Infinite	118 (98.3)	96 (80.0)

5.0	200,000	116 (96.7)	78 (65.0)
	
	1,000,000	118 (98.3)	89 (74.2)
	
	Infinite	118 (98.3)	98 (81.7)

The row with "*C*" = 2 and "Population size" = 200,000 identifies the numbers and percentages of confidence intervals containing their targets of mixture parameters, based on 10 repetitions in each of which 25 samples of size 50000 were simulated from a 4-component normal mixture with 12 parameters; results under the heading of "Bias adjustment included" are based on Equation (7) with *C *= 2, results under the heading of "Bias adjustment omitted" are based on Equation (6) with *C *= 2, and the 25 samples of size 50000 had overlap consistent with a population size of 200,000. Other rows correspond to different choices of *C *and/or population sizes.

#### c. Another example with real data

**Figure 4:**
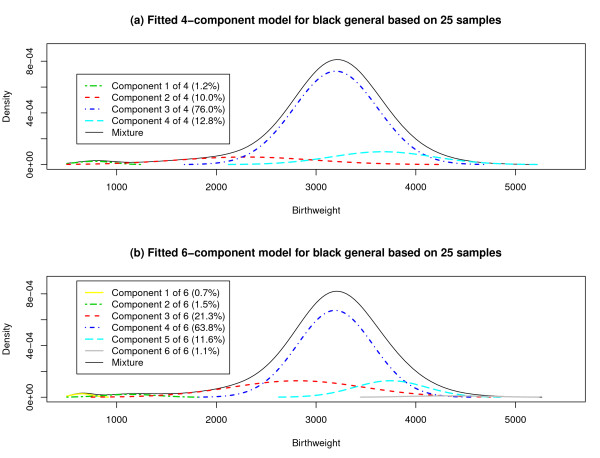
**Four- and Six-Component Mixture Models for Birthweight Distribution**. (a) A 4-component normal mixture model for birthweight distribution, with parameters estimated by combining the results for 25 samples of size 50000 from the population of black singletons in general, is shown. (b) A 6-component normal mixture model, with parameters estimated in the same manner, is depicted.

We also drew 25 samples of size 50,000 from the 1,749,827 black singletons who were born (or experienced fetal death) from 2000 to 2002, regardless of maternal smoking status. Table 6 records the frequencies with which the FLIC selected the 2- through 7-component models as well as the overall estimates of component proportions, means, and standard deviations for each of these models. The 6-component model was overwhelmingly preferred by the FLIC. Figure 4 juxtaposes the fitted 4-component and 6-component models implied by the overall estimates. The four components in the 4-component model are loosely analogous to the second through fifth components in the 6-component model, so that the main rationale for adding two more components appears to be providing a more elaborate description of the far left and right tails of the birthweight distribution.

**Table 6:** Another Example with Real Data

Model	Number of FLIC votes	Fitted Mixture Density
2 components	0	.144 *f(x;*2533,1031) + .856 *f(x;*3241,452)

3 components	0	.040 *f(x;*1300,487) + .833 *f(x;*3215,450) + .127 *f(x;*3427,656)

4 components	1	.012 *f(x;*778,186) + .100 *f(x;*2292,683) + .760 *f(x;*3198,419) + .128 *f(x;*3668,511)

5 components	1	.010 *f(x;*730,153) + .043 *f(x;*1700,490) + .655 *f(x;*3200,435) + .282 *f(x;*3289,538) + .010 *f(x;*4175,439)

6 components	22	.007 *f(x;*651,103) + .015 *f(x;*1137,273) + .213 *f(x;*2815,666) + .638 *f(x;*3191,379) + .116 *f(x;*3747,361) + .011 *f(x;*4340,409)

7 components	1	.007 *f(x;*645,101) + .013 *f(x;*1083,246) + .108 *f(x;*2415,574) + .496 *f(x;*3091,372) + .332 *f(x;*3456,383) + .038 *f(x;*4021,341) +.006 *f(x;*4613,347)

Parameters in 2-component through 7-component normal mixture models for birthweight distribution are estimated, based on 25 samples of size 50000 from the population of black singletons in general. The numbers of samples for which the FLIC preferred the various models are also recorded.

## Discussion

Our approach to modeling birthweight distribution is distinguished from previous proposals in that the data determine the number of components in the normal mixture model. We have seen that data sets of size 50,000 for white singletons born to heavily-smoking mothers typically warrant 4 components, while data sets of size 50,000 for black singletons usually demand 6 components. These results underscore the idea that a one size fits all paradigm -- whether that be a 2-component normal mixture model or even the across the board use of a 4-component normal mixture model -- may lead to unreasonable representations of birthweight distribution for some populations. Our approach, on the other hand, allows birthweight distribution to be described differently for different populations. We also note here that, although results have not been presented in this paper for a full spectrum of populations, our experience has been that data sets of size 50,000 usually call for between 3 and 6 components.

The second paper in our two-part series will elucidate the main advantage of our approach over the contaminated normal model [21,22] and the 2-component model [23], namely its greater potential to expose heterogeneity in mortality risk. By this we mean that, even at a fixed birthweight, some infants may be at higher risk than others. While such heterogeneity seems plausible, if not altogether obvious, it may not be adequately expressed by either the contaminated normal model or the 2-component model. Hence, allowing a model to have more than 2 components is not an intellectual exercise or fitting the data for the sake of fitting the data but rather a way to improve assessment of mortality.

Since gestational age is sometimes considered in tandem with birthweight [19,20], we now comment on its relation to the methodology in this two-part series.

Our approach to modeling birthweight distribution does not explicitly consider gestational age. However, our experience is that the first component typically captures most very preterm births. For instance, the birthweight distribution for white singletons with gestational ages > 37 weeks is well approximated by a 3-component model whose components resemble the second through fourth components of a 4-component model for white singletons in general.

Even so, one may be interested in extending our methodology to explicitly consider gestational age and/or other covariates. We envisage at least two possible extensions. The first would generalize the work of Fang, Stratton, and Gage [19] in which the number of components had been constrained *a priori *to two, while the second would be novel.

The first extension would be to model the joint probability density of birthweight and gestational age as a bivariate normal mixture, with the number of components determined from the data using the FLIC rather than being constrained *a priori *to two. Then, instead of estimating the mortality risk within each component as a function of birthweight only, one could estimate the mortality risk within each component as a function of both birthweight and gestational age.

The second extension would be to retain the univariate normal mixture model for birthweight distribution but create auxiliary models to relate covariates, such as gestational age, to mixture components. The appeal of this extension is that it could allow some mixture components to be placed in approximate correspondence with identifiable subpopulations.

## Conclusions

The present paper, the first in a two-part series, develops a new and flexible approach to modeling a birthweight distribution using a normal mixture model with the number of components determined from the data rather than fixed *a priori*. This approach allows the detection of heterogeneity in birthweight that cannot be found with a contaminated normal model or a 2-component normal mixture model. Unlike a contaminated normal model, our approach does not assume the existence of an interval of birthweights over which there are no compromised pregnancies. Unlike a 2-component normal mixture model, our approach does not constrain birthweights within compromised pregnancies to be normally distributed. Yet, better modeling of birthweight distribution is a means to an end, namely a greater understanding of fetal-infant mortality. The second paper in our two-part series reveals that, when coupled with methodology for estimating birthweight-specific mortality curves within each component, this paper's approach to describing a birthweight distribution can also reveal heterogeneity in mortality.

## Methods

[Additional file 1] presents technical details on our methodology and its implementation.

## Abbreviations

AIC: Akaike Information Criterion; BIC: Bayesian Information Criterion; ELBW: extremely low birthweight; EM: expectation maximization; FLIC: Flexible Information Criterion; HBW: high birthweight; MLBW: moderately low birthweight; NBW: normal birthweight; NCHS: National Center for Health Statistics; PMLR: parametric mixtures of logistic regressions; VLBW: very low birthweight

## Competing interests

The authors declare that they have no competing interests.

## Authors' contributions

RC - Concept and design, analysis and interpretation of data, drafting of the manuscript, critical revision of the manuscript for important intellectual content, statistical analysis, read and approved final manuscript. LWC - Concept and design, acquisition of data, analysis and interpretation of data, drafting of the manuscript, critical revision of the manuscript for important intellectual content, read and approved final manuscript. TL - Analysis and interpretation of data, drafting of the manuscript, critical revision of the manuscript for important intellectual content, read and approved final manuscript. RSK - Analysis and interpretation of data, drafting of the manuscript, critical revision of the manuscript for important intellectual content, read and approved final manuscript.

## Pre-publication history

The pre-publication history for this paper can be accessed here:

http://www.biomedcentral.com/1471-2393/10/37/prepub

## Supplementary Material

Additional file 1 **Technical Appendix**. Additional file 1 presents technical details on our methodology and its implementation. Click here for file
