# Supplementary material for: Thinking outside the curve, part I: modeling birthweight distribution
Source: BMC Pregnancy Childbirth. 2010 Jul 28;10:37. doi: 10.1186/1471-2393-10-37 (PMC2927479; doi:10.1186/1471-2393-10-37)
Supplement: Additional file 1 — Technical Appendix. Additional file 1 presents technical details on our methodology and its implementation. [file 1471-2393-10-37-S1.DOC]

**Additional File 1 (Technical Appendix) for**

Thinking outside the curve, part I:

modeling birthweight distribution

Richard Charnigo,^1*^ Lorie W. Chesnut,^2^ Tony LoBianco,^3^ Russell S. Kirby^4^

^1^Departments of Statistics and Biostatistics

University of Kentucky

Lexington, KY 40506-0027, USA

^2^Department of Epidemiology

University of Kentucky

Lexington, KY 40536-0003, USA

^3^Interdisciplinary Human Development Institute

University of Kentucky

Lexington, KY 40506-0051, USA

^4^Department of Community and Family Health

University of South Florida

Tampa, FL 33612, USA

* corresponding author

RC RJCharn2@aol.com

LWC lorie.chesnut@uky.edu

TB tflobi1@email.uky.edu

RSK rkirby@health.usf.edu

**I. Computational issues**

The rationale for our approach to fitting normal mixture models – i.e., beginning with the expectation maximization (EM) algorithm [31] and then shifting to the optimization (optim) procedure in version 2.3.1 of R – is as follows.

A principal advantage of EM, and our reason for starting with it, is that the likelihood increases with each iteration. Hence, EM estimates after any number of iterations are necessarily better than whatever initial values the researcher may supply [28]. Two disadvantages of EM are that: (i) EM estimates may be sensitive to initial values since the likelihood may have multiple local optima; and, (ii) EM converges very slowly [28].

In practice we find that (i) is mainly a problem for models with too many components. Fortunately, if EM leads to a local optimum that is not the global optimum for an overly complicated model, we actually benefit since information criteria then judge the overly complicated model less favorably. Still, initial values should be chosen thoughtfully.

Rather than generate initial values randomly, for reproducibility we choose them as follows: the initial proportions are taken equal and to sum to 1; the initial means are taken evenly spaced between the minimum and maximum values in the data set; and, the initial standard deviations are taken equal, their common value being the square root of the average squared deviation from a data point to the nearest initial mean.

Issue (ii) is what motivates our switch from EM to optim after at most 500 iterations of the former. The EM estimates are regarded as intermediate but not final estimates, and they serve as initial values for optim.

We simultaneously impose two sets of constraints on the final estimates from optim. The first set of constraints is that final proportions cannot be less than 0.002 or more than 1.1 minus the largest intermediate proportion (from EM), final means cannot be less than 500 grams or more than 5500 grams, and final standard deviations cannot be less than 100 grams or more than 2500 grams. The second set of constraints is that a final proportion cannot differ from the corresponding intermediate proportion by more than 0.10, a final mean cannot differ from the corresponding intermediate mean by more than 500 grams, and a final variance cannot differ from the corresponding intermediate variance by more than 1 square kilogram.

We do not start with optim because doing so would require modifying the second set of constraints to allow for greater departures from researcher-specified initial values than the departures permitted from EM-based intermediate estimates. With tighter constraints there seems to be less opportunity for non-convergence of optim.

A technique to ensure that final estimates are satisfactory is to tabulate -2 log *L*_1_ through -2 log *L_M_*. In principle they should form a monotone sequence with some sharp decreases followed by a near-plateau. Departures from such a pattern – in particular, increases from -2 log *L_m_*  to -2 log *L_m_*_+1_ for any *m* between 1 and 6 – suggest inferior local optima or non-convergence of optim. Increases of less than 6 points are unlikely to affect order selection, as the penalty terms in Equations (2) through (4) grow by at least 6 points whenever a component is added. However, being conservative, we adopt the convention that a 2-point or greater increase from -2 log *L_m_* to -2 log *L_m_*_+1_ warrants refitting the (*m*+1)-component model with tighter constraints on the final estimates. Details regarding the tightening of constraints are available on written request to the corresponding author.

**II. Assessing uncertainty in mixture parameter estimation**

a. Derivation of Equation (8)

Let *n* denote the sample size and *P* the size of the underlying population. The probability that a member of the population is not selected for any one of the *N_rep_*  samples is . Thus, the expected number of population members selected for at least one of the *N_rep_* samples is . For example, with *n* = 50,000, *P* = 202,849, and *N_rep_* = 25 the expected number of population members selected for at least one of the samples is 202,677.

Assuming that the variance of is approximately inversely proportional to the number of distinct population members in the *N_rep_* samples, the approximate factor by which the variance is inflated due to overlaps among the *N_rep_* samples is , where *nN_rep_*  is the apparent aggregate size of the *N_rep_* samples. Putting = *n / P* and taking a square root, to convert from a variance to a standard deviation, yields Equation (8).

b. Rationale for sampling with replacement

Given that the variance of is inflated by overlaps among the *N_rep_* samples, one may wonder why we do not sample without replacement. The reason is that *P* will not generally be divisible by *n*.

In our example on infants born to heavily-smoking mothers, sampling without replacement would allow at most 200,000 of the 202,849 infants to contribute to the estimation of mixture parameters at a fixed sample size of 50,000. (Of course the sample size could be changed, but then there would be difficulties in making comparisons across populations; we will elaborate on this later.) In contrast, drawing *N_rep_* = 25 samples with replacement allowed approximately 202,677 infants to contribute to the estimation of mixture parameters.

The difference of 2,677 = 202,677 – 200,000 is not dramatic, but this example is atypical in that *P* is only very slightly greater than a multiple of *n*. If *P* had been 199,999, then the difference would have been 49,848 = 199,848 – 150,000.

As is intuitively plausible, we approach a point of diminishing returns once *N*_rep_ surpasses *P / n*. In fact, / cannot shrink indefinitely as *N_rep_* increases but rather must converge to *C*_0_ .

With = .2465 as in our example on infants born to heavily-smoking mothers, the 25^th^ sample only added about 56 infants not present in the first 24 samples. The point of diminishing returns is not reached as quickly when is small. Even when is large, however, there may still be some benefit to taking *N_rep_*  large. We may be more comfortable with a 4-component model if (say) the FLIC favors it 22 out of 25 times than if the FLIC favors it 3 out of 4 times.

c. Rationale for a fixed sample size

One may wonder why we advocate taking samples of a fixed size rather than allowing sample sizes to vary across populations or, for that matter, analyzing all of the data from each population.

Fitting a finite normal mixture is computationally intensive, due to the iterative natures of EM and optim. If we are extremely patient, this alone need not discourage us from analyzing all of the data from each population. However, there is a more fundamental issue that cannot be circumvented by patience: the FLIC tends to prefer more components as the sample size increases. This occurs partly because small sample sizes provide weak signals for components with small proportions, whereas large sample sizes provide strong signals, and partly because the true distribution of birthweights in a population is not exactly a finite normal mixture. (If it were, then occasionally there should be negative numbers for birthweights!)

Although the FLIC will select the “correct” number of components with high probability when the true distribution is a finite normal mixture, the behavior of the FLIC is less well understood when the true distribution is merely well approximated by a finite normal mixture. The simulation study in Section 3a of Results provides some insights into this behavior, including the recognition that the FLIC may identify more components than desired when the sample size is very large. This is not really a weakness of the FLIC, as the BIC does the same, but rather an indication that statistical inference is delicate when the true distribution describing a scientific phenomenon is not contained in the class of statistical models under consideration – even though the true distribution may be very well approximated by one or more members of that class.

Thus, we fix the sample size to ensure that comparisons across populations are fair. For instance, a 4-component model for one population based on a sample size of 50,000 may not indicate greater heterogeneity than a 2-component model for another population based on a sample size of 10,000, but it does suggest more heterogeneity than a 2-component model for another population based on a sample size of 50,000. Note that the sample size must be fixed at a number less than or equal to the size of the smallest population in which there is interest.

d. Comparing mixture parameters within and between populations

Equation (7) can be used to make comparisons within a population, if we take to be a difference of mixture parameters rather than a mixture parameter itself. For instance, applying Equation (7) with allows us to assess whether component 3 and component 2 have different means (by seeing whether 0 is excluded from the confidence interval for ).

A comparison between populations can be made by calculating a confidence interval for – via

– + or - { + *C* }, Eq. (A)

where is a mixture parameter (such as ) for one population, is the same mixture parameter for another population, and are the corresponding estimates, and quantities with and subscripts are analogous to Equation (7) quantities with subscripts.

**III. Overall estimates and confidence intervals with smaller sample sizes**

Table A provides overall estimates and confidence intervals for parameters in a 4-component model for the birthweights of white singletons born to heavily-smoking mothers, using Equations (7) and (8) with *N_rep_* = 25 samples of size 5,000, *C*_0_ = 2.5, and = .0246 = 5,000 / 202,849. For Table B the samples are of size 10,000 and = .0493 = 10,000 / 202,849. For Table C the samples are of size 25,000 and = .1232 = 25,000 / 202,849.

Comparing Tables A through C to Table 2, we see that the confidence intervals for *p*_3_, *p*_4_, , , and narrow dramatically when the sample size increases from 5,000 to 50,000, with most of the improvement in precision coming between 5,000 and 25,000. That there is little or no improvement from 25,000 to 50,000 is not surprising. With a population size of 202,849, the expected number of population members included in *N_rep_* = 25 samples rises only 3.79%, from 195,278 to 202,677, when the sample size increases from 25,000 to 50,000.

**Tables**

Table A: Estimating Parameters in a Four-Component Mixture Model, Sample Size 5000

| Quantity | p_1_ | p_2_ | p_3_ | p_4_ |
| --- | --- | --- | --- | --- |
| [average of 25 estimates] | .009 | .168 | .742 | .081 |
| [standard deviation of 25 estimates] | .003 | .052 | .094 | .060 |
| [bias adjustment] | .002 | .034 | .076 | .061 |
| Confidence interval | (.006, .012) | (.104, .232) | (.612, .872) | (0, .177) |
| Quantity | μ_1_ | μ_2_ | μ_3_ | μ_4_ |
| [average of 25 estimates] | 875 | 2681 | 3157 | 3809 |
| [standard deviation of 25 estimates] | 130 | 199 | 35 | 187 |
| [bias adjustment] | 48 | 144 | 25 | 121 |
| Confidence interval | (753, 997) | (2422, 2940) | (3111, 3203) | (3580, 4037) |
| Quantity | σ_1_ | σ_2_ | σ_3_ | σ_4_ |
| [average of 25 estimates] | 226 | 681 | 410 | 423 |
| [standard deviation of 25 estimates] | 70 | 67 | 26 | 65 |
| [bias adjustment] | 35 | 54 | 21 | 33 |
| Confidence interval | (151, 302) | (589, 773) | (375, 446) | (353, 494) |

Parameters in a 4-component normal mixture model for birthweight distribution are estimated, based on 25 samples of size 5000 from the population of white singletons born to heavily smoking mothers. Interval estimates are constructed using Equations (7) and (8) with *C*_0_ = 2.5 and = .0246.

Table B: Estimating Parameters in a Four-Component Mixture Model, Sample Size 10000

| Quantity | p_1_ | p_2_ | p_3_ | p_4_ |
| --- | --- | --- | --- | --- |
| [average of 25 estimates] | .008 | .172 | .755 | .065 |
| [standard deviation of 25 estimates] | .002 | .059 | .076 | .032 |
| [bias adjustment] | .001 | .037 | .042 | .012 |
| Confidence interval | (.005, .010) | (.096, .248) | (.663, .847) | (.032, .099) |
| Quantity | μ_1_ | μ_2_ | μ_3_ | μ_4_ |
| [average of 25 estimates] | 825 | 2694 | 3163 | 3810 |
| [standard deviation of 25 estimates] | 104 | 173 | 19 | 118 |
| [bias adjustment] | 27 | 130 | 8 | 67 |
| Confidence interval | (730, 920) | (2451, 2937) | (3142, 3183) | (3665, 3954) |
| Quantity | σ_1_ | σ_2_ | σ_3_ | σ_4_ |
| [average of 25 estimates] | 200 | 716 | 414 | 414 |
| [standard deviation of 25 estimates] | 56 | 60 | 19 | 50 |
| [bias adjustment] | 40 | 66 | 11 | 35 |
| Confidence interval | (123, 276) | (611, 822) | (391, 438) | (346, 481) |

Parameters in a 4-component normal mixture model for birthweight distribution are estimated, based on 25 samples of size 10000 from the population of white singletons born to heavily smoking mothers. Interval estimates are constructed using Equations (7) and (8) with *C*_0_ = 2.5 and = .0493.

Table C: Estimating Parameters in a Four-Component Mixture Model, Sample Size 25000

| Quantity | p_1_ | p_2_ | p_3_ | p_4_ |
| --- | --- | --- | --- | --- |
| [average of 25 estimates] | .007 | .168 | .771 | .053 |
| [standard deviation of 25 estimates] | .001 | .034 | .036 | .010 |
| [bias adjustment] | .001 | .043 | .031 | .017 |
| Confidence interval | (.005, .009) | (.095, .242) | (.708, .834) | (.028, .079) |
| Quantity | μ_1_ | μ_2_ | μ_3_ | μ_4_ |
| [average of 25 estimates] | 811 | 2724 | 3170 | 3812 |
| [standard deviation of 25 estimates] | 57 | 104 | 7 | 53 |
| [bias adjustment] | 35 | 144 | 8 | 54 |
| Confidence interval | (724, 897) | (2487, 2962) | (3155, 3184) | (3710, 3913) |
| Quantity | σ_1_ | σ_2_ | σ_3_ | σ_4_ |
| [average of 25 estimates] | 198 | 740 | 419 | 421 |
| [standard deviation of 25 estimates] | 33 | 30 | 10 | 47 |
| [bias adjustment] | 22 | 57 | 7 | 31 |
| Confidence interval | (147, 250) | (657, 823) | (403, 434) | (348, 495) |

Parameters in a 4-component normal mixture model for birthweight distribution are estimated, based on 25 samples of size 25000 from the population of white singletons born to heavily smoking mothers. Interval estimates are constructed using Equations (7) and (8) with *C*_0_ = 2.5 and = .1232.
